# N-terminal domain of androgen receptor is a major therapeutic barrier and potential pharmacological target for treating castration resistant prostate cancer: a comprehensive review

**DOI:** 10.3389/fphar.2024.1451957

**Published:** 2024-09-18

**Authors:** Ye Chen, Tian Lan

**Affiliations:** ^1^ Department of Anesthesiology, Xi’an International Medical Center Hospital Affiliated To Northwest University, Xi’an, Shaanxi, China; ^2^ Department of Urology, Xi’an International Medical Center Hospital Affiliated To Northwest University, Xi’an, Shaanxi, China

**Keywords:** castration resistant prostate cancer, androgen receptor, N-Terminal domain, ARsplice variants, drug target

## Abstract

The incidence rate of prostate cancer (PCa) has risen by 3% per year from 2014 through 2019 in the United States. An estimated 34,700 people will die from PCa in 2023, corresponding to 95 deaths per day. Castration resistant prostate cancer (CRPC) is the leading cause of deaths among men with PCa. Androgen receptor (AR) plays a critical role in the development of CRPC. N-terminal domain (NTD) is the essential functional domain for AR transcriptional activation, in which modular activation function-1 (AF-1) is important for gene regulation and protein interactions. Over last 2 decades drug discovery against NTD has attracted interest for CRPC treatment. However, NTD is an intrinsically disordered domain without stable three-dimensional structure, which has so far hampered the development of drugs targeting this highly dynamic structure. Employing high throughput cell-based assays, small-molecule NTD inhibitors exhibit a variety of unexpected properties, ranging from specific binding to NTD, blocking AR transactivation, and suppressing oncogenic proliferation, which prompts its evaluation in clinical trials. Furthermore, molecular dynamics simulations reveal that compounds can induce the formation of collapsed helical states. Nevertheless, our knowledge of NTD structure has been limited to the primary sequence of amino acid chain and a few secondary structure motif, acting as a barrier for computational and pharmaceutical analysis to decipher dynamic conformation and drug-target interaction. In this review, we provide an overview on the sequence-structure-function relationships of NTD, including the polymorphism of mono-amino acid repeats, functional elements for transcription regulation, and modeled tertiary structure of NTD. Moreover, we summarize the activities and therapeutic potential of current NTD-targeting inhibitors and outline different experimental methods contributing to screening novel compounds. Finally, we discuss current directions for structure-based drug design and potential breakthroughs for exploring pharmacological motifs and pockets in NTD, which could contribute to the discovery of new NTD inhibitors.

## Introduction

Early detection and treatment of organ-confined prostate cancer (PCa) are associated with excellent outcomes, but treatments for advanced PCa are still largely palliative (Hamdy et al., 2023; Bill-Axelson et al., 2005; Jones et al., 2011; Wilt et al., 2012; Bill-Axelson et al., 2014; Hamdy et al., 2016; Wilt et al., 2017; de Bono et al., 2011; Parker et al., 2013; Beer et al., 2014; Sweeney et al., 2015; Mateo et al., 2015; Chi et al., 2019; de Wit et al., 2019; Smith et al., 2022; Fizazi et al., 2023). Advanced PCa is initially treated with androgen deprivation therapy (ADT) combined with androgen receptor (AR) inhibitors (ARIs), aiming at decreasing androgen concentration and antagonizing AR transactivation to blunt oncogenic effects of AR signaling (Chi et al., 2019; Smith et al., 2022; Hussain et al., 2013; Messing et al., 1999; Eisenberger et al., 1998; Crawford et al., 1989; Davis et al., 2019). However, adaptive evolution in oncogenic gene emerges which allows AR signaling to be reactivated following prolonged treatment with ADT despite ARIs based combined therapies. (Martinez-Jimenez et al., 2023; Erickson et al., 2022; Ceder et al., 2016; Gundem et al., 2015; Yuan and Balk, 2009). Generally, this dynamic evolution is achieved by the selective intervention of the androgen-dependent and independent compensatory pathways which drive the development of castration resistant prostate cancer (CRPC) (Grasso et al., 2012; Kwan and Wyatt, 2022; Mao et al., 2022; Crowley et al., 2021). Interestingly, more potent inhibitors of androgen synthesis and ARIs demonstrate benefits for CRPC, highlighting the critical role of AR signaling in the progression of CRPC (Ryan et al., 2013).

AR is a ligand-activated transcription factor which is activated by androgens, including testosterone and more active form, dihydrotestosterone (DHT) (Evans, 1988; Grino et al., 1990; Gerald and Raj, 2022). Full-length AR protein is comprised of N-terminal domain (NTD), DNA-binding domain (DBD), hinge region, and C-terminal ligand-binding domain (LBD). Of which, LDB has been the main target for ARIs, where blocking ligand binding can stop receptor activation and consequently repress AR-dependent transcription (Maylin et al., 2021). However, CRPC cells can adapt to low androgen levels through AR gene mutations and amplifications, or constitutively activate the expression of AR-regulated genes through AR spliced variants (AR-Vs) (Taplin et al., 1995; Culig et al., 1993; Visakorpi et al., 1995; Al Salhi et al., 2023). Moreover, the upregulation of coactivators and androgen-producing enzymes within tumor cells and microenvironment contribute to AR signaling persistence in CRPC (Nakamura et al., 2005). Consequently, novel AR inhibitors are constantly emerging, including LBD inhibitors (enzalutamide, apalutamide, and darolutamide), AR protein degradation enhancers (Niclosamide, Dimethylcurcumin, and UT-34), NTD inhibitors (EPIs, Sintokamides, and QW07), DBD inhibitors (Pyridium pamoate, VPC compounds, and Hairpin pyrrole-imidazole polyamides), and AR dimerization inhibitor (VPC-17005) (Scher et al., 2012; Hussain et al., 2018; Smith et al., 2018; Smith et al., 2021; Fizazi et al., 2019; Fizazi et al., 2020; Liu C. et al., 2014; Kang et al., 2023; Wang et al., 2017; Ponnusamy et al., 2019; Maurice-Dror et al., 2022; Hirayama et al., 2020; Yan et al., 2021; Peng et al., 2020; Pal et al., 2019; Butler et al., 2017; Yang et al., 2013; Dalal et al., 2018). Nevertheless, the complex and multifaceted nature of CRPC makes it challenging to identify effective drugs.

NTD is the key structural domain for AR dimerization, DNA binding as well as transcriptional regulation (Chan and Dehm, 2014). Moreover, this domain is abundantly post-translationally modified and acts as a hub for interactions with many other coregulatory proteins (Cucchiara et al., 2017; Sawada et al., 2023). Interestingly, in truncated forms of AR which is devoid of the LBD, NTD can fold in functional state and act as a constitutively fully active form, suggesting a central role of the NTD as a transcriptional driver (Haile and Sadar, 2011). However, NTD is largely unstructured and described as having limited stable secondary structure which can be induced by interactions with binding partners to increase α-helical content and thereby conforms to a molten-globule-like conformation referred to as ‘collapsed disordered’ (Sadar, 2020). Therefore, intrinsic disorder and high dynamic conformation prevent 3-dimension structural determination, and as such, the molecular mechanism behind NTD function remains elusive. In this review, we initially focus on the structure-function relationship of NTD, then highlight the pharmaceutical potential of current NTD inhibitors and the strategies focused on screening compounds. In the end, we propose current design direction and outline the potential breakthrough for discovering novel NTD-inhibitors.

### Sequence-structure-function relationship of N-Terminal domain

#### The sequence of mono-amino acid in NTD endues the nature of intrinsic disorder

**FIGURE 1:**
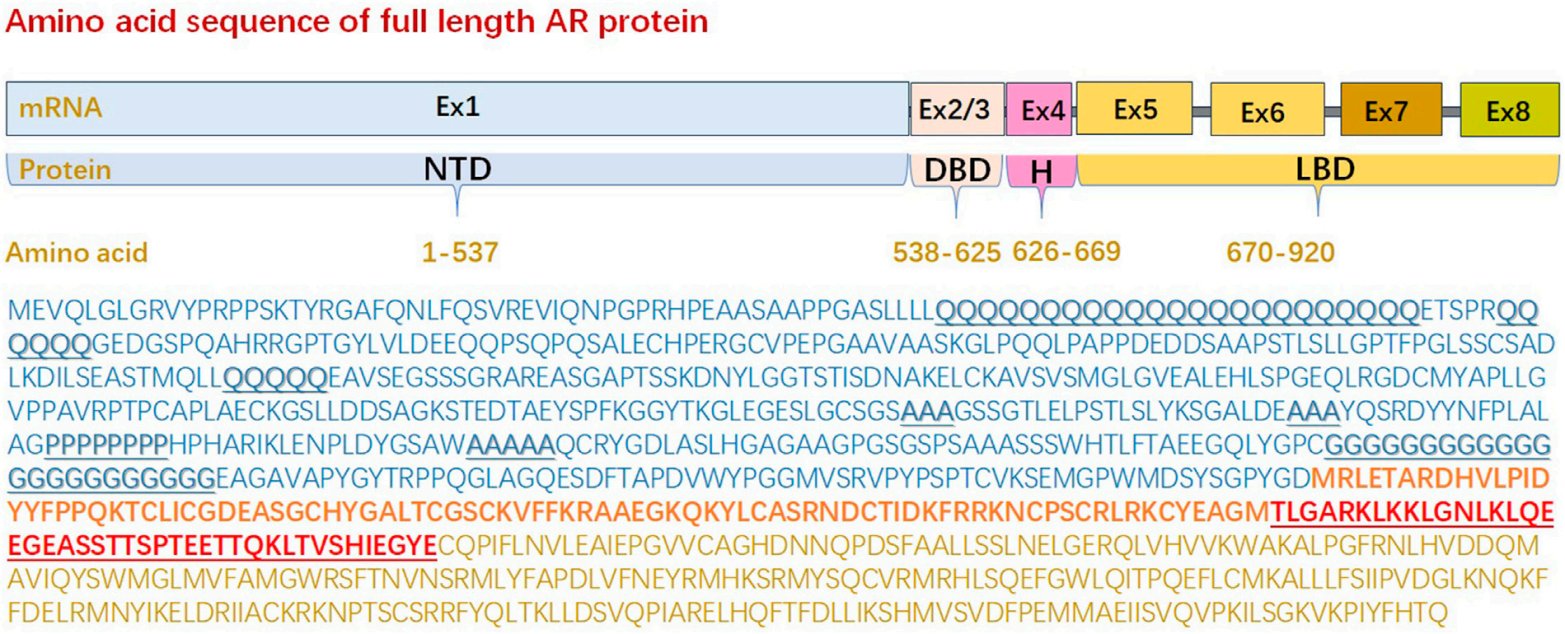
Amino acid sequence of full length androgen receptor protein. Full-length androgen receptor (920aa) is comprised of an intrinsically disordered N-terminal domain (1-537aa), a folded DNA binding domain (538-625aa), a variable hinge region (626-669aa), and folded C-terminal ligand binding domain (670-920aa) that contains the ligand-binding pocket. The sequence of N-terminal domain lacks order-promoting amino acids, such as Ile (I, <1%), Leu (L, 8.7%), Asn (N, 1.1%), Val (V, 1.6%), Phe (F, 1.3%), Cys (C, 2.0%), Tyr (Y, 1.3%), Trp (W, <1%), most of which are hydrophobic, whereas N-terminal domain are rich in amino acids that are either polar or charged. Moreover, N-terminal domain harbors several repeat regions of glutamine (aa 58–80/86–91/195–199), proline (aa 374–381), alanine (aa 331–333/356–358/400–404), and glycine (aa 451–473), of which polyQ1 and polyG are polymorphic. (AR: androgen receptor, DBD: DNA binding domain, Ex: exon, LBD: ligand binding domain, NTD: N-terminal domain).

In NTD, the abundance of charged and polar amino acids, coupled with a lack of hydrophobic ones, results in disorder-promoting forces (interaction with surrounding water) that overwhelm ordering and compacting forces (resulting from hydrophobic interaction), leading to disorder. Moreover, in full-length AR (920aa), NTD harbors several repeat regions of glutamine (polyglutamine tract or polyQ1/Q2/Q3), proline (ployP), alanine (polyA), and glycine (polyG) (Faber et al., 1989; Palazzolo et al., 2008; Blessing et al., 2015; Lieberman and Fischbeck, 2000; Grigorova et al., 2017), of which polyQ1 and polyG are polymorphic (Figure 1). The variable length of polyQ1 tract is the most studied among these repeat regions, and Its length impacts AR solubility and transcriptional activity. AR with short polyQ1 tracts tends to have increased transcriptional activity whereas a longer repeat region has less activity (Tut et al., 1997). Moreover, the longer polyQ1 sequences hinder nuclear localization in the absence of hormone and increase the propensity for formation of AR-containing puncta in the nucleus of cells treated with DHT (Buchanan et al., 2004). Helicity of the polyQ1 tract is stabilized by H-bonds between the side chains of glutamine and the main carbonyl groups and helical structure of the tract correlates with its length. Changes in conformation would presumably impact NTD interactions with other proteins and thereby modulate its transcriptional activity in addition to its solubility and propensity to form fibrils. Moreover, AR liquid-liquid phase separation behaviors are reported related to transcriptional activity and antiandrogen efficacy. The NTD can undergo liquid-liquid phase separation *in vitro*, with longer polyQ1 sequences phase separating more readily (Roggero et al., 2022).

Intrinsically disordered proteins (IDPs) affect myriad biological pathways and biomolecular assemblies ranging from organelle formation, forming liquid droplets via phase separation, chromatin modulators, and stress tolerance to gene transcription (Handa et al., 2023; Wu et al., 2022). Unlike folded proteins, NTD populates a conformational ensemble of rapidly interconverting structures in solution. NTD also remains highly dynamic while interacting with proteins or compounds. The conformations of NTD samples a large number of topological arrangements, and structures in conformational ensembles have little-to-no structural similarity to one another. NTD possesses sequence elements with elevated secondary structure populations relative to random-coil distributions. This confers a limited amount of local order to conformational ensembles, but the number of thermally accessible orientations of sidechain and backbone pharmacophores can remain combinatorically large. These local orders are generally not possible to represent even small sequence segments of NTD by a single dominant conformation. NTD are, therefore, not suitable targets for conventional structure-based drug-design methods that require the existence of an ordered binding site, and the general “druggability” of NTD sequences remains uncertain.

### Activation function-1 within disordered N-Terminal domain is essential for AR transactivation

**FIGURE 2:**
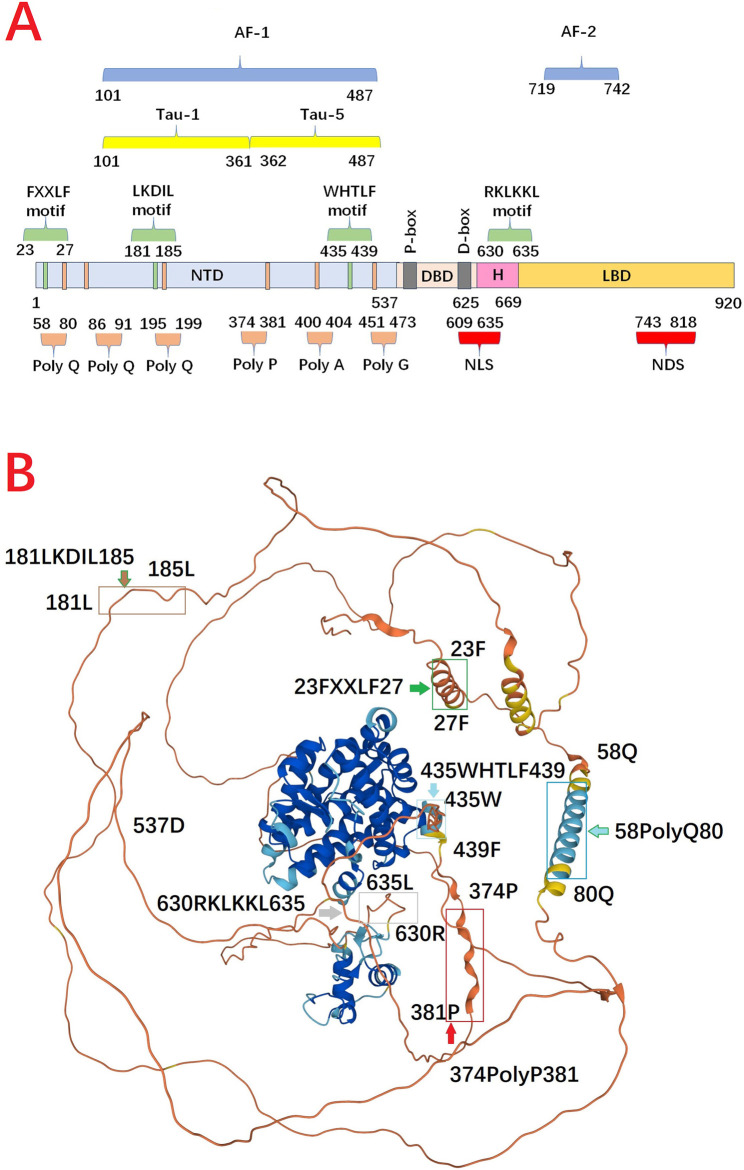
**(A)** Functional domain and motif in full length androgen receptor protein. **(B)** 3D structure of androgen receptor from AlphaFold Protein Structure Database. N-terminal domain harbors several repeat regions for glutamine (polyQ, aa 58–80), and proline (ployP, aa 374–381). Meanwhile, N-terminal domain contains at least three distinct regions proposed to generate amphipathic-helices, including 23FxxLF27, 181LKDIL185, and 435WxxLF439, which can interact with the hydrophobic groove in ligand binding domain. DNA binding domain has a compact, globular structure in which three substructures can be distinguished: two zinc clusters and a more loosely structured carboxy terminal extension. Hinge region can be defined as the fragment between the last α-helix of the DNA-binding domain and the first α-helix of the ligand binding domain. The 630RKLKKL635 motif, plays a central role in controlling AR activity, not only because it acts as the main part of the nuclear translocation signal, but also because it regulates the transactivation potential and intranuclear mobility of the receptor. ligand binding domain is comprised of 11 α-helices which encompass a ligand-binding pocket. When androgen binds, there is a shift in conformation to reposition helix 12 over the ligand-binding pocket to create the activation function-2 surface for interaction with coactivators. Alpha Fold produces a per-residue model confidence score (pLDDT) between 0 and 100 (Dark Blue >90, Blue >70, Yellow >50, Orange <50). Some regions below 50 pLDDT may be unstructured in isolation (https://alphafold.ebi.ac.uk/entry/D3YPP9) (Varadi et al., 2024) (AF-1: the activation function-1, AF-2: activation function-2, DBD: DNA binding domain, H: Hinge region, LBD: ligand binding domain, NDS: nuclear degration sequence, NLS: nuclear localization sequence, NTD: N-terminal domain, Tau-1: transcriptional activation unit-1, Tau-5: transcriptional activation unit-5).

AR is unique from other steroid hormone receptors in that the activation function-2 (AF-2) domain in LBD has no identified transcriptional activation unit (Tau). Specifically, transactivation function of AR is largely dependent on the ligand-independent activation function-1 (AF-1) domain, which is located in NTD (Davey and Grossmann, 2016). In AF-1, transcriptional activation unit-1 (Tau-1, 101–361 aa) is estimated to have 13% helical secondary structure, of which a large number are acidic amino acids but these helical structures can increase upon interaction with a binding partner. The core domain mediating AF-1 transcriptional activity has been mapped to a discreet 181LKDIL185 motif within the NTD (Callewaert et al., 2006). Moreover, transcriptional activation unit-5 (Tau-5, 362–487 aa) in AF-1 is responsible for the majority of constitutive transcriptional activity within the NTD, and is mediated through the core sequence 435WHTLF439. Tau-5 is not acidic which harbors three different amino acid stretches, including ployP (aa 374–381), polyA (400–404), and polyG (aa 451–473). The size and location of this activation function domain in the human AR is variable, being dependent on the promoter context and the presence or absence of the LBD (Jenster et al., 1995) (Figure 2).

### N-terminal domain is the primary site for recruiting transcriptional regulators

There are nearly 200 known coregulators that can activate or repress the expression of AR target genes, and many of these interactions between AR and coregulators occurs within NTD (van de Wijngaart et al., 2012; Heemers and Tindall, 2007; Culig and Santer, 2012). The CREB-binding protein (CBP) and p300 are important regulatory proteins due to their ability to acetylate histone and non-histone proteins to modulate transcription (Eckner et al., 1994; Chrivia et al., 1993; Dancy and Cole, 2015). CBP/p300 not only enhance AR-mediated transactivation, but also facilitate the androgen-dependent interaction between the N-terminal domains and C-terminal domains (N/C interaction) (Ban et al., 2021). AR recruits CBP/p300 through an indirect interaction mediated by the Steroid Receptor Co-activator proteins (SRCs) which interact with LBD, then recruit CBP/p300 to the AR activation complex (Powell et al., 2004). SRC proteins interact with LBD via an LXXLL motif in the SRC receptor interaction domain. Interestingly, SRC-1 interacts with AF-1 domain in NTD, which appears functionally more important than SRC-1/LBD interaction for AR-mediated transcriptional activation (Powell et al., 2004). Interaction between SRC proteins and CBP/p300 is essential for the histone acetylation of AR target genes, including prostate-specific antigen (PSA) encoding gene, KLK3 (Kallikrein Related Peptidase 3) (Shang et al., 2002). Simultaneously, the co-activator-associated arginine methyltransferase 1 (CARM1) is recruited to AR target genes along with CBP/p300, which methylates specifically H3R17 and R26 to activate gene expression (Baek et al., 2006). Recently, cryo-electron microscopy (cryo-EM) study demonstrates that androgen-activated AR homodimer binds the enhancer of the KLK3 gene, and each AR monomer directly interacts with p300 via NTD and small portions of the LBD. In concordance with structural data, immunoprecipitation experiment also verify the NTD is required for the direct AR-p300 interaction (Yu et al., 2020).

### The conformation shift of N/C interaction

There Newly synthesized AR which is present in the cytoplasm is associated with major molecular chaperones, including the heat shock protein 70 (Hsp70), Hsp40, Hsp90, and choline kinase alpha (CHKA) (Dong et al., 2019; Estebanez-Perpina et al., 2005; Hildenbrand et al., 2011; Asim et al., 2016). In the absence of androgen, this apo-receptor which is in a complex with chaperone proteins facilitates AR’s solubility and retains a high-affinity conformation for the androgenic hormones. Androgen binding results in conformational shifts of AR, which causes release of chaperones from AR. Meanwhile, LBD undergoes a conformational change which presents a hydrophobic groove to interact with the LXXLL motif presenting in coactivators, such as SRCs (Powell et al., 2004; Heery et al., 1997). SRCs, in turn, recruit other coactivators that are capable of promoting histone modifications or chromatin remodelling, such as p300/CBP, to stimulate transcriptional initiation (Zhong et al., 2014; Kamei et al., 1996; Onate et al., 1995)

**FIGURE 3:**
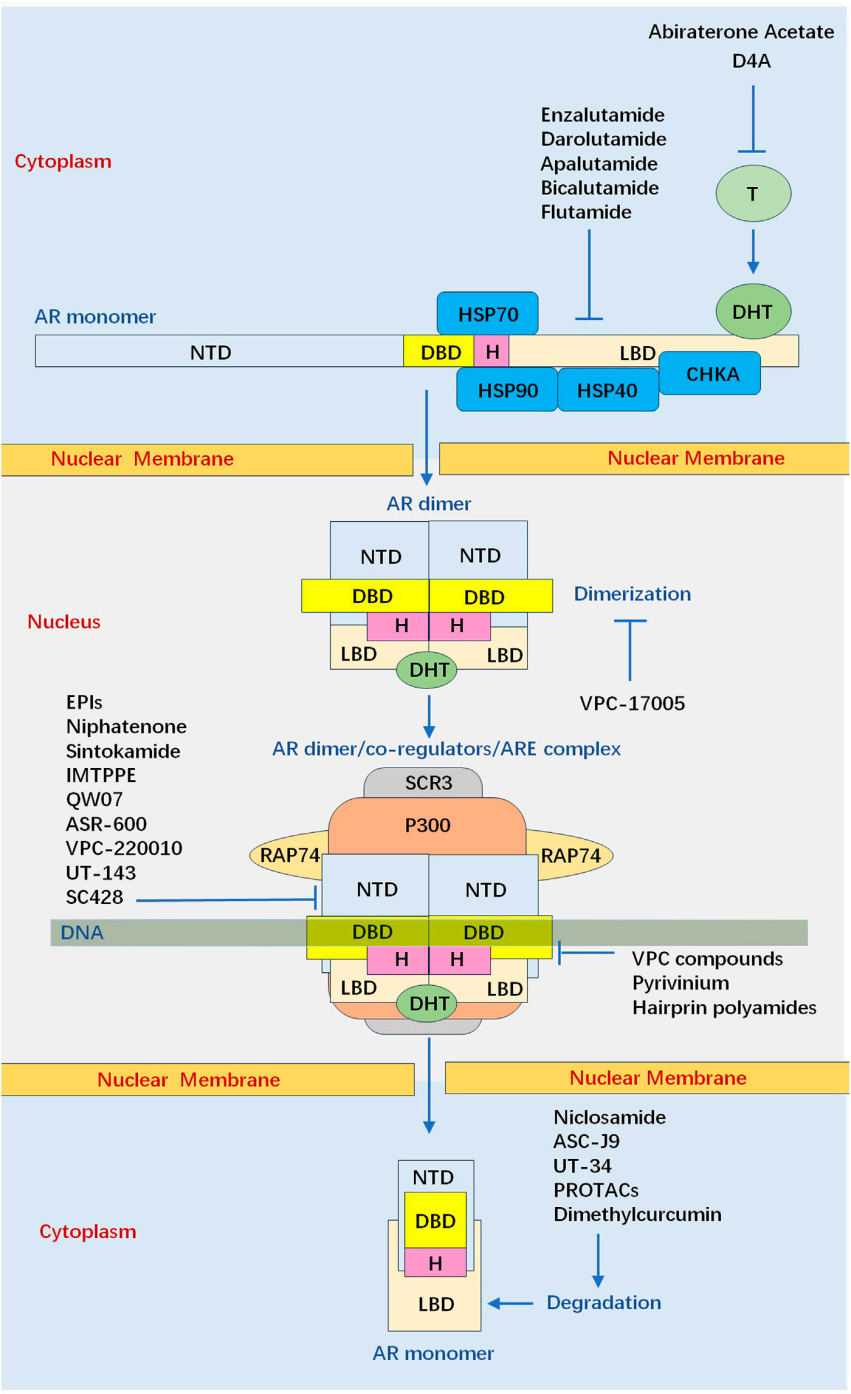
Summary of current and experimental inhibitors that target the androgen receptor signalling axis via different mechanisms. Abiraterone acetate inhibit the production of DHT. First-generation (Bicalutamide, Flutamide) and second-generation (enzalutamide, apalutamide, and darolutamide) LBD-targeting ARIs competitively inhibit the DHT binding to ligand binding domain. VPC-17005 Binds L594-S613 of AR-DNA binding domain D-box to inhibit dimerization of androgen receptor. Emerging small molecules (EPIs, Niphatenone, Sintokamide, IMTPPE, QW07, ASR-600, VPC-220010, UT-143, SC428) have been able to bind the N-terminal domain of the androgen receptor and thus suppress transactivation of androgen receptor target genes. VPC com-pounds, Pyrivinium, Hairprin polyamides bind the DNA binding domain thus preventing interaction with DNA, inhibit androgen receptor dimerization so that it cannot interact with DNA. Niclosamide, ASC-J9, UT-34, PROTACs, Dimethylcurcumin upregulate AR protein degradation to reduce the abundance of the androgen receptor protein. (AR: androgen receptor, ARI: androgen receptor inhibitor, CHKA: choline kinase alpha, DBD: DNA binding domain, DHT: dihydrotestosterone, Hsp: heat shock protein, LBD: ligand binding domain, NTD: N-terminal domain, PROTAC: proteolysis-targeting chimera, SRCs: steroid receptor co-activator proteins).

After being activated in cytoplasm, AR is transferred into the nucleus where the androgen/AR complex dimerizes and binds to androgen response elements (AREs) (Wong et al., 1993; van Royen et al., 2012). N/C interaction is rapidly initiated in the cytoplasm after hormone binding as an intramolecular interaction, and is followed by an intermolecular N/C interaction in the nucleus, contributing to receptor dimerization (van Royen et al., 2007). Generally, AR dimerization follows a unique head-to-head and tail-to-tail manner (Yu et al., 2020), and the strong amino N/C interaction between 23FXXLF27 motif in NTD and binding groove in LBD is necessary to mediate transcriptional activity for full length AR (Steketee et al., 2002; Thompson et al., 2001).For AR dimer, NTD contains at least three distinct regions proposed to generate amphipathic-helices, including 23FxxLF27, 181LKDIL185, and 435WxxLF439, which can interact with the AF-2 hydrophobic groove (Callewaert et al., 2006; He et al., 2000). This dynamic change reduces solubility and improves affinity to DNA. Moreover, N/C interaction reduces the dissociation rate of bound androgen and slows the degradation rate of the carboxyl-terminal binding domain. The N/C interaction occurs preferentially in the transferring AR, which can protect the coactivator binding groove against unfavourable protein-protein interactions. When AR dimer binds to DNA, the N/C interactions are largely lost, and the interactions between AR and coregulators preferentially occur (van Royen et al., 2007; Ozgun et al., 2021) (Figure 3).

### The modeled tertiary structure of N-Terminal domain

Cryo-EM analysis of the full-length AR in complex with an interacting coregulatory partner, suggests NTD is a disordered conformation that surrounded LBD (Yu et al., 2020). In cryo-EM structure of DNA-bound AR/SRC-3/p300 complex, two NTDs surround the LBD dimer so that only a small portion of the LBD is exposed. LBD and DBD form a tight dimerization interface at the center, and both NTDs connect to each other. Consequently, AR dimerization follows a unique head to head and tail to tail manner. Moreover, only one SRC-3 is observed in this complex and p300 directly interacts with AR, without bridging through SRC-3. The AF-1 domain of AR-NTD is responsible for p300 recruitment and p300 largely contacts the NTDs of both AR monomers. Unlike p300, SRC-3 interacts with the NTD of one AR monomer (Figure 3).

To explore how cross-talking between structured domains and disordered regions regulates the complicated cellular functions an combination of molecular dynamics (MD) simulations and circuit topology (CT) analysis is employed to identify the tertiary structure of NTD. Topological mapping of the dynamics reveals a traceable time-scale dependent topological evolution of conformation shift (Sheikhhassani et al., 2022). After MD simulations visual examination shows that the initial conformation of NTD could undergo an extensive structural change. Importantly, two disjoint regions in the global 3D shape are emerged: an extended N terminal sub-region in NTD (NR, residues 1–224), and a C terminal sub-region in NTD (CR, residues 225–538). Furthermore, a segment as NTD-CR core is denoted which is differentiate from the rest of the NTD-CR, named CR shell. Interestingly, the core included the well-known Tau-5 that is significantly more compact than NR. Therefore, NTD adopts highly dynamic loopy conformations with two identifiable regions accompanying with distinct topological make-up and dynamics, and this loopy conformation consists of the NR and the CR, which carries a dense core. Meanwhile, NR adopts different positioning with respect to the CR and forms a cleft that can partly enclose LBD. Furthermore, data suggest a model in which dynamic NR and CR can compete for binding to the DBD of AR, thereby regulating the accessibility of its DNA-binding site.

### Screen AR-NTD inhibitors to offer potential for pharmaceutical development

#### Discovery of AR variants promotes the exploration of NTD-inhibitors

**FIGURE 4:**
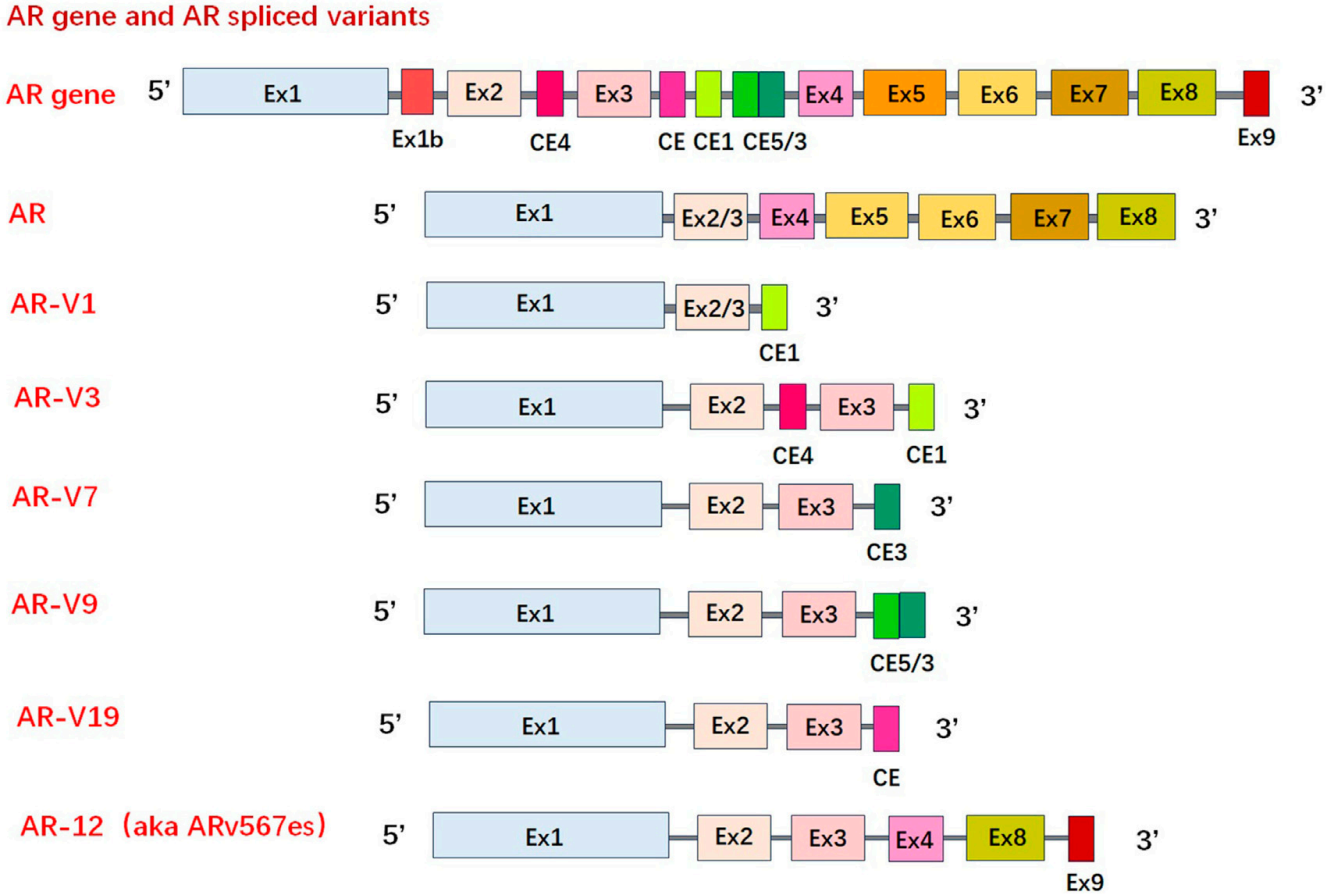
Schematic of mRNA of wild type androgen receptor/androgen receptor variants. Over 20 androgen receptor variants have been identified. Truncated androgen receptor variants form due to alternative splicing and/or structural gene rearrangements of the androgen receptor gene. (AR-V: androgen receptor variant, Ex: exon).

The constitutive activation of AR-Vs are associated with resistance mechanisms of CRPC (Antonarakis et al., 2014). Over 20 AR-Vs have been identified in human PCa cells, xenografts, and clinical specimens (Katleba et al., 2023). Generally, AR-Vs have been detected in normal prostate and treatment-naive PCa, however, they are much more common in CRPC, leading to the hypothesis that AR-Vs are created in higher quantities due to treatment pressure (Chen and Lan, 2021). Many variants cannot be bound by LBD-targeting drugs since they do not contain the LBD. AR-V7 is the most common of the constitutively active AR-variant, with AR-V567es and AR-V3 also in this category (Hu et al., 2009). Other variants, such as AR-V9, tend to be conditionally active, with its transcriptional activity depending on the cellular context (Hu et al., 2011; Wustmann et al., 2023). These truncated AR-Vs form due to alternative splicing and/or structural gene rearrangements of the AR gene. For example, AR-V7 mRNA is spliced at the alternative 3′ splice site next to the cryptic exon 3, as opposed to the 3′ splice site by exon 4, this, therefore translates into a C-terminal truncated form of AR protein (Liu LL. et al., 2014) (Figure 4). As the transcriptional activity of AR largely resides in NTD an inhibitor targeting NTD should block the transcriptional activities of all AR species. Moreover, AR-NTD has little sequence similarity (<15%) to other steroid hormone receptors, such as estrogen receptor, and thereby NTD is considered as a drug target that could be highly specific to block AR signalling (McEwan et al., 2000).

At present, X-ray crystal structures of LBD and DBD have been solved separately (Nadal et al., 2017; Ohta et al., 2011; Matias et al., 2000; Sack et al., 2001; Shaffer et al., 2004). However, the intrinsic disorder of NTD poses a challenge not only for experimental analysis of the conformation but also for computational modelling of the chain due to the size of the conformation space and lack of stable folds. For example, the state-of-the art artificial intelligence-based prediction approaches fail to identify the conformation of NTD. Therefore, current NTD inhibitors are often discovered using cell-based assay for compounds screening. Meanwhile, misfolding of the NTD is also implicated in PCa and Kennedy’s disease (Thomas et al., 2004). Yet our knowledge of NTD structure is limited to primary sequence information of the protein chain and a few functionally secondary structure motifs.

**FIGURE 5:**
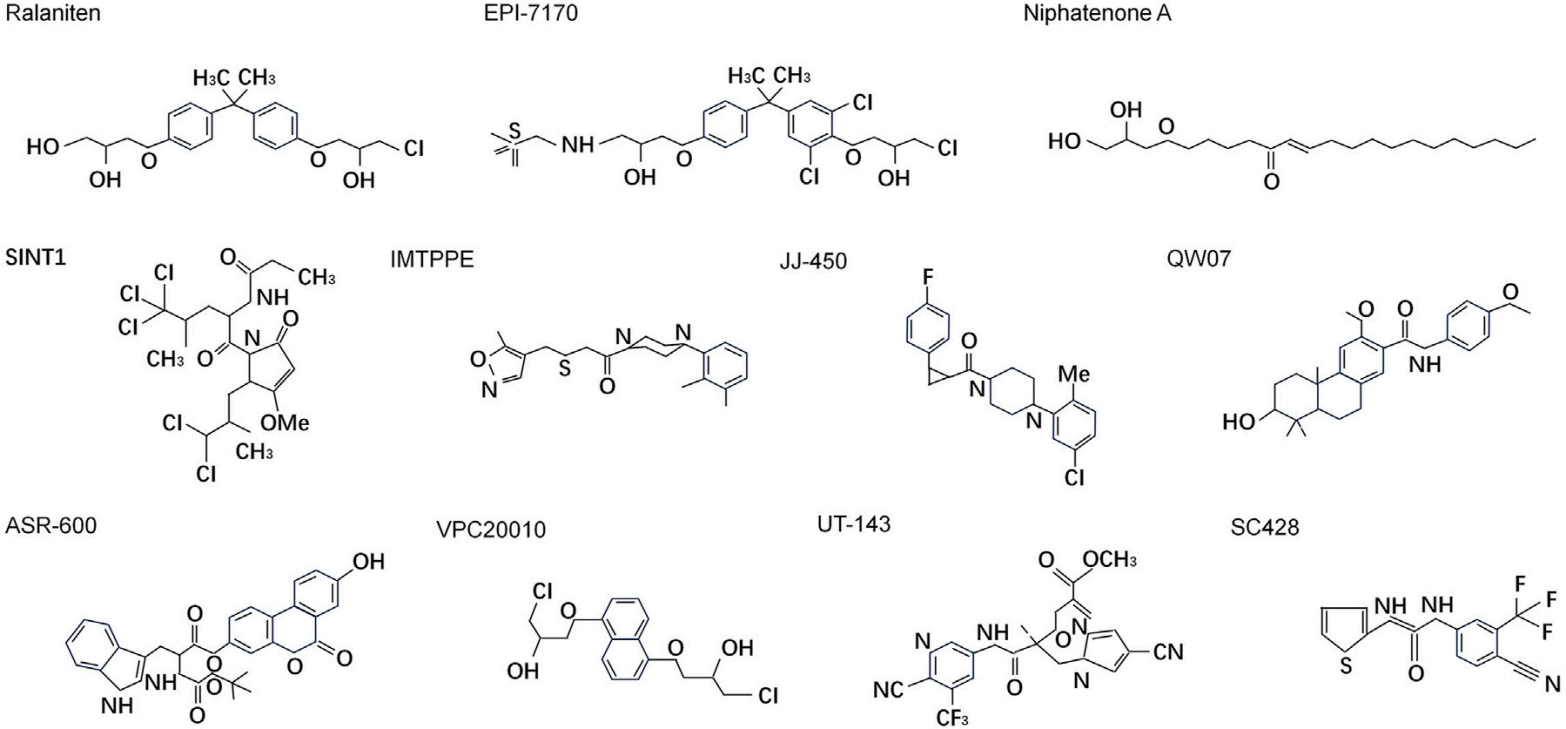
Chemical structures of small molecules directly targeting N-terminal domain of androgen receptor.

Despite being disordered, it is reasonable to assume that at least some residues in the disordered protein regions would adopt certain arrangements to form functional conformation. Meanwhile, the disordered states and structures of IDP/Rs could be dictated by the protein sequence and thus “intrinsic” to the encoding sequence. Cysteines form covalent bonds as sulfide bridges to stabilize protein structure when the environment is oxidizing, but under reducing conditions the bridges are broken and usually the protein will be disordered and inactivated (Fraga et al., 2014; Meszaros et al., 2018). Moreover, the presence of aromatic residues within IDRs may reveal a MoRF. MoRF are defined as “short, interaction-prone segments that undergo disorder-to-order transitions upon specific binding, representing a specific class of intrinsically disordered regions that exhibit the molecular recognition and binding characteristics” (Cheng et al., 2006). MoRF regions are of substantial interest in drug development due to their potential for compounds binding (Yan et al., 2016). Starting in 2003, several small molecule libraries were tested by Sadar lab aimed to discover small molecules that specifically binds to NTD. Over 52,000 compounds from the NCI or Chembridge libraries were tested with little to no hits. Fortunately the natural compounds libraries provided approximately 30 hits from unique extracts which were further fractionated to purification and isolation of the active compounds. Three of these compounds (ralaniten, sintokamides, and niphatenones) that directly interact with NTD have been discovered (Maurice-Dror et al., 2022; Hirayama et al., 2020; Yan et al., 2021; Meimetis et al., 2012; Banuelos et al., 2014). The remaining active scaffolds and extracts (IMTPPE/JJ450, QW07, VPC-220010, ASR-600, etc.) continue to be optimized and characterized by employing different experimental methods (Peng et al., 2020; Masoodi et al., 2017; Chandrasekaran et al., 2023) (Table 1) (Figure 5).

**TABLE 1:** Summarizing current inhibitors targeting N-terminal domain of androgen receptor.

Screening strategies	Original compounds	Derivatives	AR signaling impacts	Cellular impacts	Therapy phase
Cell-based high throughput assays	Bisphenol A Diglycidic Ether	EPI-002 EPI-056 EPI-045 EPI-7170 EPI-7386	1. Inhibits AR activity regardless of DHT 2. Steroid receptor specificity 3. Blocks N/C interaction 4. Interaction with AF1 region 5. Blocks interactions with co-regulator 6. Do not alter intracellular localization of AR	Inhibit proliferation 1. LNCaP 2. MDA PCa2B 3. 22RV1	1. Inhibits xenograft of: LNCaP Models 2. Phase I clinical trial NCT02606123 NCT04421222
	Niphatenone	Niphatenone B	1. Inhibits AR activity regardless of DHT 2. Interaction with AF1 region on AR and GR 3. Blocks N/C interaction 4. Do not alter intracellular localization of AR	Inhibit proliferation 1. LNCaP	
	Sintokamide	Sintokamide A Analogue 76	1. Inhibits AR activity 2. Steroid receptor specificity 3. Do not alter intracellular localization of AR 4. Interaction with AF1 region on AR 5. Inhibits constitutively active of AR-V567es	Inhibit proliferation 1. LNCaP	Inhibits xenograft of: LNCaP Models
	IMTPPE	JJ-450 (+)-JJ-74–138	1. Directly bind to AR 2. Inhibits AR activity 3. Steroid receptor specificity 4. Inhibits constitutively active of AR-V7 5. Reduce AR level in the nucleus	Inhibit proliferation 1. LNCaP 2. C4-2 3. 22Rv1	Inhibits xenograft of 22Rv1 Models LNCaP Models
Proteolysis-targeting chimera	BWA-522		1. Degradate AR-FL and ARV7	Inhibit proliferation 1. LNCaP 2. VCaP 3. 22Rv1	Inhibits xenograft of LNCaP Models
	ITRI-90		1. Effectively degrade AR 2. Inhibits AR activity	Inhibit proliferation 1. LNCaP 2. C4-2 3. 22Rv1 4. VCap	Inhibits xenograft of 22Rv1 Models
Bispecific antibodies	3E10-AR441		1. Enters LNCaP cells 2. Accumulates in nucleus 3. Inhibits signaling of AR and AR-V7	Inhibit proliferation 1. C4-2	
Modelling structure-based pharmacophore	QW07	Tricyclic diterpenoids	1. Inhibits AR activity 2. Binds to AR-NTD directly	Inhibit proliferation 1. LNCaP 2. C4-2 3. 22Rv1 4. VCap	Inhibits xenograft of 22Rv1 Models VCaP Models
Blocking AR-Vs signalling	Urolithin A	ASR-600	1. Target and inhibit AR and AR-V7 expression 2. Downregulate AR by ubiquitin signaling 3. Specifically target AR signaling 4. Binds to AR-NTD directly	Inhibit proliferation 1. LNCaP 2. C4-2 3. 22Rv1 4. VCap	Inhibits xenograft of 22Rv1 Models VCaP Models
	Biaryl isoxazole compound 7	Biaryl isoxazole compound 16	1. Inhibition of AR-Vs activity		
	SC428		1. Directly binds to the NTD (aa 507–531) 2. Inhibits activity of AR-V7 and ARv567es 3. Impaire AR/AR-V7 nuclear translocation 4. Do not affect AR-V7 levels 5. Disrupted AR-V7 homodimerization	Inhibit proliferation 1. LNCaP 2. 22Rv1 3. VCap	Inhibits xenograft of 22Rv1 Models
	UT-34/UT-21c	UT-143	1. Covalently bind to AF-1 (C406 and C327) 2. Reduce the activity of AR and AR-V7 3. Interfer liquid–liquid phase separation 4. Degrade AR 5. Change AF-1 Conformation	Inhibit proliferation 1. LNCaP 2. 22Rv1	Inhibits xenograft of LNCaP Models
Structured-based virtual screening	VPC-3033	VPC-2055 VPC-220010	1. Without AR degradation 2. Inhibiting AR-V7 activity 3. Specificity of AR 4. Disrupt interactions of co-regulators	Inhibit proliferation 1. LNCaP 2. 22Rv1	
	EIQPN		1.Inhibiting AR activity 2.Bind to AF-1 3.Decreases AR levels	Inhibit proliferation 1. LNCaP 2. C4-2 3. 22Rv1	Inhibits xenograft of 22Rv1 Models
Targeting associated co-regulators	Compound named 154		1. Inhibits AR/STAT3 transcriptional activity 2 .Inhibits levels of AR 3. Inhibits levels of ARv567es and AR‐V7 3. Bind to AR-NTD 4. Disrupt interaction of AR and STAT3 5. Do not reduce AR nuclear translocation	Inhibit proliferation 1. LNCaP	
	Thio-2	A4B17	1. Inhibits binding of BAG1L to AR 2. Inhibiting AR-V7 activity	Inhibit proliferation 1. LNCaP 2. LNCaP95 3. 22Rv1	
	HBC		1. Inhibits AR activity regardless of DHT 2. Abrogat AR interaction with Calmodulin 3. Bind exclusively to CaM 4. Suppress phosphorylation of AR (Serine81)	Inhibit proliferation 1. LNCaP 2. C4-2 3. 22Rv1	Inhibits xenograft of C4-2b Models
	BCT		1. Directly bind to HSP90 2. Disrupt interaction with AR/AR-V7 3. Degradation of AR and AR-V7 4. Inhibiting AR/AR-V7 activity	Inhibit proliferation 1. LNCaP 2. C4-2B 3. C4-2B-MDVR 4. VCaP 5. 22RV1	Inhibits xenograft of 22Rv1 Models
	Y08197		1. Inhibit CBP/EP300 bromodomain 2. Inhibiting AR activity	Inhibit proliferation 1.LNCaP 2. C4-2B 3. VCaP 4. 22RV1	
	JQ1		1. Bind to NTD of BRD4 2. Disrupt interaction of BRD4 to AR-NTD 3. Inhibiting AR activity	Inhibit proliferation 1. LNCaP 2. VCaP 3. 22RV1	Inhibits xenograft of VCaP Models

### Screen small-molecule inhibitors by high throughput cell-based assays

#### EPI derivatives

The original EPI compounds are isolated from the marine sponge which are most likely of industrial origin based upon its structural resemblance to Bisphenol A Diglycidic Ether (BADGE) (Hutson, 1998; Andersen et al., 2010). BADGE is a harmless metabolite of bisphenol A without estrogenic or androgenic effects, which cannot be converted by biological systems back to the estrogenic bisphenol A (Terasaki et al., 2006; Nakazawa et al., 2002). Importantly, at high daily doses, BADGE and its chlorohydrins are not carcinogenic nor genotoxic (Yang et al., 2022; Suarez et al., 2000; Sueiro et al., 2001; Chang et al., 2014). EPIs and analogues inhibit the transcriptional activities of full-length AR as well as AR-Vs (Sadar, 2020; Myung et al., 2013). Moreover, the inhibition of AR transcriptional activity is specific as EPI analogues has no effect on the activities of other steroid receptors (Andersen et al., 2010). Generally, unlike some ARIs, which can cause AR to translocate to the nucleus and bind to DNA binding sites of target genes, EPI analogues do not induce nuclear translocation in the absence of androgen. Interestingly, EPIs block AR binding to the promoters and enhancers of target genes to decrease expression of these genes in response to androgens (Andersen et al., 2010). Furthermore, EPI analogues inhibit the N/C interaction of AR which is required for androgen-dependent transactivation of AR (Andersen et al., 2010). Meanwhile, EPI analogues can also block the interactions of CBP and RAP74 with NTD, which is essential for AR transcriptional activity (Andersen et al., 2010).

EPI-001 has two chiral centres and is a mixture of four stereoisomers, EPI-002 (2R, 20S), EPI-003 (2S, 20R), EPI-004 (2R, 20R), and EPI-005 (2S, 20S). EPI-002, the single stereoisomer of EPI-001, is assigned the generic name Ralaniten by the United States Adopted Names (USAN) council, and a new stem class “-aniten” created based upon its novel mechanism of action that distinguishes it from the C-terminal LBD nonsteroidal antiandrogens with the stem name “lutamide”. Phase I clinical trial that investigated the safety profile of ralaniten acetate (EPI-506), the prodrug of EPI-002, was initiated in 2015 (NCT02606123) (Maurice-Dror et al., 2022). The poor pharmacokinetic profile of EPI 506 ultimately prevented further advancement into Phase II clinical trial. As EPI-506 was subject to glucuronidation by UDP-glucuronosyltransferase (UGT2B) enzymes resulting in loss of potency (Obst et al., 2019). Afterwards, the detection of glucuronidated metabolites of ralaniten in the serum of patients promoted the development of next-generation ralaniten analogues. EPI-045, a derivative of ralaniten, was resistant to UGT2B mediated glucuronidation and showed increased potency as compared to ralaniten against a background of elevated UGT2B activity (Obst et al., 2019). Another derivative, EPI-7170, has approximately 10 times better potency for inhibiting AR transcriptional activity compared to ralaniten (Zhu et al., 2022; Banuelos et al., 2020). Combination of EPI-7170 and enzalutamide resulted in synergistic inhibition of proliferation of enzalutamide-resistant cells (Hirayama et al., 2020). Moreover, by tethering EPI-002 and different classes of E3 ligase ligands, a small molecular proteolysis-targeting chimera (PROTAC) compounds targeting NTD were obtained. For example, a representative compound, BWA-522, which induced degradation of both AR-FL and AR-V7 suppressed the expression of AR downstream related proteins (Zhang et al., 2023). ITRI-PROTAC compounds mechanistically degrade AR-FL and AR-V proteins via ubiquitin-proteasome system, and inhibit cell proliferation. The compounds also significantly inhibit enzalutamide-resistant growth of CRPC cells. In CWR22Rv1 xenograft model, ITRI-90 displays a pharmacokinetic profile with decent oral bioavailability and strong antitumor efficacy (Hung et al., 2023).

#### Niphatenone

Niphatenones are originally isolated in active extracts from Niphates digitalis marine sponge, and shows strong activity in cell-based assay, which is designed to screen antagonists of AR (Meimetis et al., 2012). Assay-guided fractionation shows niphatenones A and B, two new glycerol ether lipids, are the active components of the extracts (Meimetis et al., 2012). Niphatenone B and its enantiomer can block androgen-induced proliferation of LNCaP cells but has no effect on the proliferation of PC3 cells that do not express AR (Meimetis et al., 2012). Interestingly, click chemistry analysis shows that niphatenone B binds covalently to AF1 region of NTD (Meimetis et al., 2012). Both enantiomers of niphatenone B have similar IC50 values of 6 µM for inhibiting the full-length AR in a functional transcriptional assay, and the (S)-niphatenone have significantly better activity against the NTD compared to (R)-niphatenone (Banuelos et al., 2014). Moreover, niphatenones inhibit functional transcription activation of AR-Vs, and these compounds do not affect the transcriptional activity of progesterone receptor (Banuelos et al., 2014). Meanwhile, Niphatenones can block N/C interactions of AR without altering either AR protein levels or intracellular localization in response to androgen (Banuelos et al., 2014). As niphatenone decreases glucocorticoid receptor (GR) transcriptional activity by a mechanism involving covalent binding to GR AF-1 further development of these compounds is halted (Banuelos et al., 2014).

#### Sintokamide

The chlorinated peptides sintokamides A to E are isolated from the specimens of the marine sponge Dysidea, of which Sintokamide A (SINT1) binds AF-1 domain in NTD and specifically attenuates transcriptional activities of both AR and constitutively active AR-Vs (Sadar et al., 2008). *In vivo*, SINT1 reduces the expression of an AR-regulated gene, PSA and causes regression of CRPC xenografts (Sadar et al., 2008; Banuelos et al., 2016). Combination of SINT1 with ralaniten has an additive effects on AR transcriptional activities, which implies that the SINT1 binding site on AF-1 is unique from ralaniten (Banuelos et al., 2016). SINT1 binds a recombinant AF-1 protein at residues 143–486, therefore it is hypothesized that SINT1 binds closer to the N-terminus of the AF-1, potentially overlapping or within Tau-1 and thus inhibiting AR transactivation (Banuelos et al., 2016). Moreover, SINT1 has shown specificity for NTD binding which does not interact with other steroid hormone receptors as well as the LBD of AR (Banuelos et al., 2016). As SINT1 has a short half-life cell-based transcriptional activity assay is used to monitor the potency of analogues of Sintokamide (Yan et al., 2021). Results shows that the chlorine atoms on the leucine side chains are essential for potent activity (Yan et al., 2021). Analogues missing the nonchlorinated methyl groups of the leucine side chains (C-1 and C-17) are just as active and in some cases more active than the natural products (Yan et al., 2021). Analogues with the natural R configuration at C-10 and the unnatural R configuration at C-4 are most potent, and replacing the natural propionamide N-terminus cap with the more sterically hindered pivaloylamide N-terminus cap leads to an enhanced potency (Yan et al., 2021). As the tetramic acid fragment and the methyl ether on the tetramic acid fragment are essential for activity the structure-activity relationship optimized analogue 76 is more selective, easier to synthesize, more potent, and presumes to be more resistant to proteolysis than the natural sintokamides (Yan et al., 2021).

#### IMTPPE

2-[(isoxazol-4-ylmethyl)thio]-1-(4-phenylpiperazin-1-yl)ethanone (IMTPPE) is identified from National Institutes of Health Library of 219,055 compounds. IMTPPE is capable of inhibiting AR transcriptional activity and nuclear AR level in AR-positive PCa cells (Masoodi et al., 2017). IMTPPE also inhibits the transcriptional activity of a mutant AR variant lacking the LBD, indicating that inhibition of AR is independent of the LBD (Masoodi et al., 2017). A novel analogue of IMTPPE, JJ-450, has been investigated with the evidence for its direct and specific inhibition of AR transcriptional activity (Yang et al., 2020). JJ-450 can block AR recruitment to androgen-responsive elements and suppress AR target gene expression (Yang et al., 2020). JJ-450 can also inhibit AR-V7 transcriptional activity and target gene expression as well as the growth of AR-V7 positive CRPC cells, 22Rv1 (Yang et al., 2020). The AR point mutant, ARF876L, can be stimulated, instead of inhibited, by enzalutamide, thus contributing to enzalutamide resistance. In contrast, JJ-450 inhibit both wild-type (WT) AR and ARF876L transcriptional activity to a similar extent. Moreover, JJ-450 retards nuclear import of both AR and ARF876L (Yang et al., 2020). A analogues of JJ-450, (+)-JJ-74–138, is more potent than JJ-450 in the inhibition of androgen-independent AR activity in enzalutamide-resistant LN95 cells (Cole et al., 2022). Further studies show that (+)-JJ-74–138 inhibits PSA expression in all tested CRPC cells and the proliferation of enzalutamide-resistant AR-positive LN95 and 22Rv1 cells at low dosages, but not AR-negative PC3 and DU145 cells (Cole et al., 2022).

### Bispecific antibodies targeting NTD are developed *in vitro* experiments

Two synthetic peptides corresponding to potentially antigenic sites (aa 201–222 and aa 301–320) located within the NTD in 900aa full-length AR are used as immunogens. This approach successfully generated two antibodies against the peptide homologous to a AR fragment flanking the AF-1 sequence, which specifically stain the nuclei of glandular epithelial cells in frozen sections of human prostate tissue implying aa 201–222 and aa 301–320 might be two referable sites for protein binding (van Laar et al., 1989). Generally, antibodies bind cell surface antigens or extracellular molecules as they cannot penetrate the cell membrane. mAb 3E10, derived from an anti-DNA autoantibody from lupus nephritis-infected mice (Weisbart et al., 1990), however, can penetrate mammalian cells rapidly. Moreover, uptake of mAb 3E10 is dependent on the equilibrative nucleoside transporters 2 (ENT2), a nucleotide salvage receptor. Bispecific antibodies targeting the NTD are developed using 3E10 as a scaffold and combining single-chain variable fragments (scFv) additions joined by linkers. AR441 is a known anti-AR antibody which immunogen is the synthetic peptide corresponding to AR aa 302–318 (STEDTAEYSPFKGGYTK). AR441 is used as the scFv component due to its epitope being located in NTD creating the antibody BiAb 3E10-AR441. This BiAb antibody is able to inhibit the AR transcriptional activity by binding to NTD of full-length AR and AR-Vs, and reducing transactivation of target genes leading to inhibition of androgen-dependent cell growth *in vitro* (Goicochea et al., 2017). At present, whether 3E10-AR441 can penetrate cancer cells *in vivo* remains be determined.

### Small-molecule compounds screened by modelling structure-based pharmacophore

#### QW07

Using luciferase reporter assay which contains aa 1–558 of AR and the yeast transcription factor GAL4 (AR1-558-Gal4BD), the small molecule compound, named QW07, is demonstrated as an NTD-specific antagonist with the potential to inhibit both canonical and variant-mediated AR signalling pathway (Peng et al., 2020). Surface plasmon resonance (SPR) analysis and function evaluation show that QW07 binds to NTD directly, blocks the transactivation of NTD, disrupts the interactions between co-regulatory proteins and ARE, inhibits the expression of genes downstream of AR and inhibited PCa cells growth *in vitro* and *in vivo* (Peng et al., 2020). As tricyclic aromatic diterpenoid QW07 has recently been established as a potential N-terminal AR antagonist, the structure–activity relationship of tricyclic diterpenoids and their potential to suppress AR-positive PCa cell proliferation are further explored (Sekhon et al., 2023). Dehydroabietylamine, abietic acid, dehydroabietic acid, and their derivatives are selected, which have a similar pharmacophore structure as QW07. Twenty diterpenoids are prepared for the evaluation of their antiproliferative potency on AR-positive PCa cells (LNCaP and 22Rv1) using AR-null cells (PC-3 and DU145) as comparisons (Sekhon et al., 2023). Data indicates that six tricyclic diterpenoids possesses greater potency than enzalutamide towards AR-positive cells, LNCaP and 22Rv1. Moreover, four diterpenoids are more potent than enzalutamide against 22Rv1 cells (Sekhon et al., 2023) in which the optimal derivative possesses greater potency (IC50 = 0.27 µM) and selectivity than QW07 (Sekhon et al., 2023).

### Novel NTD-binding compounds are discovered attributing to blocking AR-V7 signalling

#### ASR-600

Urolithin (UroA), a dietary gut microbiota-derived metabolite of ellagic acid, shows exert anti-cancer effects on many cancer types (Totiger et al., 2019). UroA also inhibits the proliferation of both androgen-dependent (LNCaP) and androgen-independent (C4-2B) PCa cells (Dahiya et al., 2018). ASR-600, an analog of Urolithin A (Uro A), can effectively suppress the growth of AR positive CRPC cells without the effect in AR negative cells (Chandrasekaran et al., 2023). Biomolecular interaction assays including molecular docking studies, differential scanning fluorimetry and nuclear magnetic resonance (NMR) spectroscopic studies reveal that ASR-600 binds to NTD, which is further confirmed by immunoblot and subcellular localization studies (Chandrasekaran et al., 2023). Moreover, increased ubiquitin expression is observed in ASR-600 treated C4-2B and 22Rv1 cells. Molecular studies also suggest that ASR-600 promotes the ubiquitination of AR and AR-V7 resulting in the inhibition of AR signalling (Chandrasekaran et al., 2023). Microsomal and plasma stability studies suggested that ASR-600 is stable, and its oral administration inhibits tumor growth in castrated and non-castrated mice with CRPC xenograft (Chandrasekaran et al., 2023).

#### Biaryl isoxazole compound 16

Using functional cell-based assay biaryl isoxazole compound 7 is identified as a weak binder of AR splice variants (AR-vs). Library of 50 biaryl analogues are synthesized, and their biological activities are assessed in VCaP cell-based luciferase reporter gene assay. For reference, enzalutamide and EPI-001 are also examined for their effect against luciferase activity. In this assay, enzalutamide was very potent, with an IC50 of 0.34 μM, while EPI-001 had an IC50 of 37.4 μM. The structure-activity relationship study reveals that indazole analogue 16 exhibits increased potency over the progenitor compound 7 (IC50 = 0.12 μM). This represents a 60-fold increase in potency over compound 7 and compares favourably with enzalutamide (IC50 = 0.34 μM). Furthermore, compound 16 displays a promising intrinsic clearance in human liver microsomes and a half-life of 54 min. The plasma protein binding of 16 is determined and shows a free fraction of 6.9%, and no inhibition of CYP450 is observed. Moreover, pharmacokinetic profile is obtained in male CD-1 mice. Following oral dose at 10 mg/kg, a mean half-life of 4 h is observed along with adequate plasma exposure (Cmax = 195 ng/mL) and measurable exposure (AUC = 173 h ng/mL) with an oral bioavailability (F) of 16% (Henry et al., 2023).

#### SC428

SC428 is a lead compound to develop AR-V7 inhibitors which reveals a clear structure–activity relationship against AR-V7. For AR-V7, NTD might be the only targeting domain of SC428, which has been assessed from three complementary aspects: Direct binding of SC428 to AR-NTD is confirmed by SPR analysis, SC428 gain the ability to inhibit the IRF3 transcriptional activity when IRF3 transactivation domain is replaced by AR-NTD, and SC428 lose inhibition against full-length AR when NTD in AR is replaced by the transactivation domain of VP16 transcription factor (Yi et al., 2023). In addition, SC428 is selective towards NTD as SC428 being inactive against GR. Interestingly, SC428 has a significant antiproliferative effect against multiple ENZ-resistant cellular models, including 22Rv1, LNCaPAR-V7, and LNCaP-ARF877L. SC428 also shows antitumor activity against AR-V7 high-expressing tumors in intact mice as well as in castrated mice, demonstrating its therapeutic potential for men with CRPC that have suffered relapse from current AR targeting agents. Furthermore, another lead compound, named SC912, is discovered which binds to full-length AR as well as AR-V7 through NTD (Yi et al., 2023). The binding site of this small molecule relies on the amino acids 507–531 in NTD. SC912 also disrupts AR-V7 transcriptional activity, impairs AR-V7 nuclear localization and DNA binding, and suppresses the proliferation of AR-V7 positive CRPC cells (Yi et al., 2024).

#### UT-143

AF-1 protein can form molecular condensates by liquid–liquid phase separation (LLPS), and exhibit characteristics of intrinsic disordered protein, such as rapid intracellular mobility, coactivator interaction, and euchromatin induction. Selective AR degraders (SARDs) can bind the carboxy-terminal ligand binding domain and markedly reduce the activity of wildtype and splice variant isoforms of AR at sub-micromolar doses. Interestingly, two synthesized molecules (UT-34 and 21c) in SARD program can degrade AR by binding to AF-1 domain and possess properties, such as NTD binding, long half-life, low clearance in metabolism assays, and optimum pharmacokinetic and pharmacodynamic properties (Ponnusamy et al., 2019). Utilizing UT-34 and 21c as starting point, a library of covalent molecules, including UT-143, is synthesized. The LLPS and other characteristics of NTD domain can be reversed by UT-143 which covalently bind to C406 and C327 in AF-1 region of AR (Thiyagarajan et al., 2023). Interfering with LLPS formation by UT-143 can result in chromatin condensation and dissociation of AR-V7 interactome, all culminating in a transcriptionally incompetent complex. Biochemical studies further suggest that C327 and C406 in the AF-1 region are critical for condensate formation and UT-143s irreversible AR inhibition. Moreover, UT-143 possesses drug-like pharmacokinetics and metabolism properties and inhibits PCa cell proliferation and tumor growth (Thiyagarajan et al., 2023).

### Novel NTD-targeting compounds identified by structured based virtual screening

#### VPC-220010

Three million compounds from the ZINC database are docked into two selected crystal structures of the AR using Glide SP program. Two AR crystal structures (PDB code:2PNU.pdb and 3L3X.pdb) from the Protein Data Bank are prepared for docking (Li et al., 2013). Using the synergetic combination of virtual and experimental screening, a number of new 10-benzylidene-10H-anthracen-9-ones are discovered that not only effectively inhibits AR transcriptional activity, but also induces almost complete degradation of AR (Li et al., 2013). Of these 10-benzylidene-10H-anthracen-9-one analogues, a lead compound (VPC-3033), is identified that shows strong androgen displacement potency, effectively inhibits AR transcriptional activity, and possesses a profound ability to cause degradation of AR. Notably, VPC-3033 exhibits significant activity against PCa cells that have already developed resistance to the second-generation antiandrogen enzalutamide. Interestingly, one of these compounds, VPC-2055, is effective without any signs of AR degradation. Structural comparison between VPC-2055 and EPI-001 reveals that these two compounds share a chemically reactive moiety of 1-chloropropan-2-ol marked by a rectangular frame (Ban et al., 2021). Doxycycline-inducible AR-V7 transcriptional activity assay in PC3 cells confirms that VPC-2055 is indeed active against this truncated variant. Moreover, VPC-2055 could effectively inhibits the proliferation of 22RV1, an ARV7-driven cell line. Therefore, using VPC-2055 as a starting point to expand the naphthalene series, 110 novel compounds are synthesized and tested. A new compound, VPC-220010, inhibits AR-mediated transcription of full length AR and truncated variant AR-V7, downregulates AR response genes, and selectively reduces the growth of both full-length AR- and truncated AR-dependent PCa cell lines (Ban et al., 2021). Meanwhile, VPC-220010 disrupts interactions between AR and known coactivators and coregulatory proteins, such as CHD4, FOXA1, ZMIZ1, and several SWI/SNF complex proteins suggesting that VPC-220010 is a promising small molecule that could be further optimized into effective NTD inhibitor (Ban et al., 2021).

#### EIQPN

Through AR structure-based virtual screening using the FlexX docking model, 54 compounds are selected and further screened for AR antagonism via cell-based tests. One compound, 6-[6-ethoxy-5-ispropoxy-3,4-dihydroisoquinolin-2 [1H)-yl]-N-[6-methylpyridin-2-yl] nicotinamide (EIQPN) did not bind to AR-LBD unlike conventional AR antagonists. EIQPN interacts with the AF-1 domain and blocks androgen-independent activity. Moreover, EIQPN robustly decreases the protein levels of AR and variants in prostate cancer cells by inducing AR protein degradation. *In vitro*, EIQPN inhibit the androgen-independent proliferation of various AR-positive prostate cancer cells, and block the tumor growth of androgen-independent prostate cancer cells in xenograft mouse models, indicating EIQPN serves as a potential therapeutic agent for CRPC (Tran et al., 2020).

### Blocks NTD activities by targeting NTD-associated co-regulators

The ability for NTD to regulate gene expression depends on interactions with other co-regulators, and this interaction of NTD and partners shift the ensemble to a different conformation which may yield varied secondary structure. Through screening of around 600 natural compounds in prostate tumorigenesis model, potential STAT3 signalling inhibitors are examined for effects on AR signalling. A small molecular compound named 154 exhibits dual effects on IL6/STAT3 and AR pathways. Moreover, this compound is identified as an antagonist of the AR-NTD by disrupting protein-protein interactions between STAT3 and the AR NTD without reducing AR nuclear translocation (Hua et al., 2018). Another small molecule, 2-(4-fluorophenyl)-5-(trifluoromethyl)-1,3-benzothiazole (A4B17), can inhibit the interaction of BAG1L and AR-NTD, and attenuate BAG1L mediated AR-NTD activity. *In vitro*, A4B17 downregulates AR target gene expression, and inhibits proliferation of AR-positive prostate cancer cells (Kuznik et al., 2021). Calmodulin (CaM) can bind to AR-NTD and regulates AR activity. Hydrazinobenzoylcurcumin (HBC), which binds exclusively to CaM, can inhibit AR activity by abrogating AR interaction with CaM, suppressing phosphorylation of AR, and block the binding of AR to androgen-response elements. Moreover, HBC can be readily absorbed and inhibit the growth of xenografted CRPC tumors in nude mice (Wu et al., 2015).

Using cell-based reporter assay system, bruceantin (BCT) is identified as a highly potent inhibitor by suppressing the transcriptional activity of AR-FL/AR-V7 and growth of CRPC cells. Mechanistically, BCT disrupts the interaction of HSP90 with AR-FL/AR-V7 by directly binding to HSP90, leading to degradation of AR-FL/AR-V7 through the ubiquitin-proteasome system (Moon et al., 2021). CBP and its homolog EP300 are highly expressed in CRPC. Y08197, a novel 1-(indolizin-3-yl) ethanone derivative dose-dependently inhibit the CBP bromodomain with an IC50 value at 100 nM. In CRPC cells, treatment with Y08197 markedly inhibit the expression of AR regulated genes and cell growth (Zou et al., 2019). JQ1, a selective small-molecule inhibitors that target the amino-terminal bromodomains of BRD4, has been shown to exhibit anti-proliferative effects in CRPC xenograft mouse models. BRD4 physically interacts with the NTD of AR, and JQ1 can potently abrogate BRD4 localization to AR target loci and AR-mediated gene transcription (Asangani et al., 2014). Taken together, these studies provide a novel strategy for the concerted blockade of oncogenic drivers in CRPC.

### Current directions for the structure-based drug design of NTD inhibitors

#### Affirming NTD inhibitors specifically bind to endogenous AR

EPI-001 interacts with the AF-1 domain in NTD, inhibits protein-protein interactions between AR and coregulators, and reduces the interaction between AR and AREs (Brand et al., 2015). In topological dynamics model, more than 90% of interacting residues between EPI-001 and AR are located in the Tau-5 regions of NTD (residues 362–487), which suggests the high affinity of EPI-001 to this key functional motif (Sheikhhassani et al., 2022). NMR analysis also suggests that residues 354–448 are essential for EPI-001 binding (De Mol et al., 2016). Direct interaction of other EPI analogues with the AF-1 domain of NTD is further shown with recombinant protein in a cell-free assay by fluorescence emission spectroscopy (Sadar, 2011) and Click-chemistry probes (Myung et al., 2013). Due to the sensitivity of IDR conformations to environment and protein-protein interactions, proof that EPI binds endogenous AR in living cells is important to ensure that studies with the recombinant protein in cell-free assays are not artifactual. The first demonstration of direct binding of EPIs to the endogenous IDR of NTD is reported in living cells using both Click-chemistry probes and the radio-labelled analogues (Myung et al., 2013). *In vivo*, radio-labelled EPI analogue is injected into mice carrying both AR-positive xenograft and AR-negative xenograft. Only AR-positive tumors accumulates the radioactive compound thereby reaffirming the specificity of EPI to AR-NTD as well as providing proof-of-concept of the potential to image tumors using EPI compounds.

### Elucidating binding mechanism responsible for covalent attachment

EPI-002 and EPI-7170 both possess a chlorohydrin group that has been shown to be weakly reactive with Tau-5, and it is hypothesized that covalent attachment of EPI compounds to Tau-5 might be required for its biological activity. Non-covalent binding might therefore be the first step in AR inhibition which localizes reactive ligands to specific cysteines in NTD and increase their rate of covalent attachment. The atomically detailed binding mechanisms reveals that higher affinity with non-covalent binding of EPI-7170 to Tau-5 substantially increases the proximity of the EPI-7170 chlorohydrin group to the reactive thiol of residue cysteine 404, which is relative to the proximity of the EPI-002 chlorohydrin and cysteine 404 thiol groups. It is hypothesized that the non-covalent binding of EPI-7170 and EPI-002 would increase the local concentration of near weakly reactive cysteines in Tau-5, driving covalent attachment and potentially further stabilizing the formation of transcriptionally inactive compact helical states (Zhu et al., 2022).

### Exploring the specifical interacting-pocket in NTD

NMR data reveals EPI-001 and its stereoisomers directly bind to three regions within Tau-5, which is the region interacting with RAP74, supporting earlier studies that show EPIs disrupting this interaction (Andersen et al., 2010). EPIs do not bind linear amino acid sequence but rather bind three regions independently within the residues 343–448 in NTD. Moreover, all three regions must simultaneously be present for binding, corresponding to regions 343–373, 393–416 and 428–448 (De Mol et al., 2016). This suggests that EPIs bind to a conformation having a pocket, or that EPIs induce formation of a conformation to create a pocket. It also means that EPI-001 interacts with an ensemble of conformations of AF-1 adopting a partially folded structure, and binding of EPI to Tau-5 can yield a transcriptionally dead conformation that cannot interact with transcriptional coregulators.

### Providing details for dynamic binding mechanisms

All-atom MD simulation studies can provide support for different dynamic binding mechanisms between IDPs and small molecule ligands. Generally, ligands populate a broad distribution of binding modes that confer little-to-no detectable ordering relative to the apo forms of IDP (Lohr et al., 2022). In some cases, a series of small molecule ligands bind specifically to the C-terminal region of IDPs with differing affinities without significantly altering the conformational ensembles of the apo and bound states (Robustelli et al., 2022). This study proposes that the specificity and affinity of these ligands is conferred through a so-called dynamic shuttling mechanism where a ligand rarely forms multiple specific intermolecular interactions simultaneously and instead transitions among networks of spatially proximal interactions. In this mechanism, differences in the affinity and specificity of ligands are attributed to the complementarity of the orientations of protein and ligand pharmacophores in a dynamic IDP ensemble without evoking the notion of an ordered binding site. In other cases, the conformational ensembles of IDPs can undergo an entropic expansion upon ligand binding, where interactions with a small molecule can increase the number of conformations significantly populated by an IDP. Moreover, small molecule ligands can also cause a population shift among existing IDP conformations, drive the compaction of monomeric disordered state proteins upon binding or drive the formation of soluble oligomeric states (Zhao et al., 2021). The variety of binding mechanisms observed suggest that small molecules affect the conformational ensembles of IDPs in a system-dependent manner (Heller et al., 2018; Heller et al., 2015). However, yet we do not know how these novel NTD-inhibitors alter the conformational ensembles of NTD. Recently, MD simulation studies elucidates detailed binding mechanism of the ralaniten (EPI-002) and analogs (EPI-7170) (Zhu et al., 2022). The strongest interactions of EPI-002 with Tau-5 are corresponding to three regions with transiently populated helices, termed R1, R2, and R3 (De Mol et al., 2016). Moreover, these bound states remains dynamic and samples a heterogeneous ensemble of binding modes. A network of interactions that more effectively stabilize these collapsed helical conformations is identified in the EPI-7170 bound ensemble. These results suggest that EPI compounds inhibit the activity of AR by inducing the partial folding of molecular recognition elements in the Tau-5 domain into compact helical states and preventing interactions between AR and the transcriptional machinery required for elevated AR transactivation.

### Testing NTD inhibitors in clinical trials

Ralaniten acetate (EPI-506) is the first drug to be tested in clinic trial (NCT02606123) (Maurice-Dror et al., 2022). 28 patients with progressed metastatic CRPC were enrolled in phase 1 open-label study with seven dose cohorts, ranging from 80–3,600 mg. The primary outcome was the safety and tolerability, and the secondary objectives included maximal tolerated dose (MTD), pharmacokinetic profile, and antitumor efficacy. Signs of efficacy were showed by reduction of serum PSA in some patients receiving higher doses, in spite of not achieving steady-state plasma concentrations (Cmin), of what would be required for optimal therapeutic concentrations based upon *in vitro* data (25 μM). Patients who received the highest dose (3,600 mg/daily) had trough levels of approximately 0.5μM, which is 50X lower than the 25 μM required for optimal activity of ralaniten *in vitro*, and 48 to 58-fold lower than steady-state Cmin for enzalutamide and its active metabolite respectively.

Several patients received EPI-506 for more than 1 year with stable disease. Dose limited toxicities (DLTs) occurred in four patients, including elevated amylase (Grade 4); abdominal pain (Grade 3); elevated alanine transaminase (ALT) and aspartate transaminase (AST) (Grade 3); nausea (Grade 2), and vomiting (Grade 1), which resulted in drug intake of <75% of the expected dose during the DLT assessment period. The drug was considered “well-tolerated” but due to poor pharmacokinetics there was excessive pill burden. Analysis of the plasma metabolism of ralaniten acetate showed that ralaniten was both oxidized and glucuronidated. Finally, this study was terminated prior to reaching the MTD due to poor oral bioavailability. Nevertheless, this phase 1 clinic trial established the safety of EPI-506 and provides proof of concept for targeting NTD. In 2020, another phase 1, open-label study was conducted aiming to evaluate the safety, pharmacokinetics, and anti-tumor activity of a second-generation EPI compounds, EPI-7386 (NCT04421222). Recruited CRPC patients received an oral dose of EPI-7386 ranged from 200mg to 2400 mg daily with an estimated completion date in the year of 2026.

### Potential breakthroughs for discovering novel NTD-inhibitors

#### Identifying short linear motifs to design peptide-drugs

The primary function of IDRs is to bind partners using short linear motifs (SLiMs) with 3–15 residues (van der Lee et al., 2014). Until now, more than 100,000 SLiMs are identified in the human proteome, indicating that IDP interactions are widespread in diverse biological processes and diseases (Kumar et al., 2022). Moreover, flanking regions outside of the SLiMs can make nonspecific contacts with partners, thereby increasing binding affinity. Approach to designing inhibitors is to use SLiM fragment as a template. AR-NTD is essential for SRC-3/p300 recruitment, and transcription is activated when NTD binds SRC-3/p300. Peptide fragments derived from NTD can be used as inhibitors of the interaction between NTD and SCR-3/p300. Introduction of non-canonical amino acids and cyclization of NTD-derived peptide would further increase its affinity for SRC-3/p300. Moreover, peptidomimetic compounds have also been used as inhibitors that bind IDP partners. As IDRs do not adopt a specific structure, MD simulations have been performed to sample conformational ensembles of IDRs, which are then used to identify hotspots for binding, followed by virtual screening of a large number of compounds and peptide-drugs. Currently, the computational design of IDR-binding proteins and the rational design of IDR peptides that regulate the formation of biomolecular condensates have been extensively studied (Wu et al., 2023; Qian et al., 2022), and these approaches based on identifying short linear motifs will contribute to NTD drug discovery.

### Search for recurring conformation to explore transient binding pockets

For the drugs which target disordered proteins, the mechanism of action varies from binding to specific hydrophobic sites along the sequence to binding to multiple conformations and modifying the conformational ensemble. Generally, modification of the conformational ensemble with small molecule drugs could be of two different kinds. Either these drugs can force the protein into acquiring new conformations, or they can modify the conformational space by increasing the weight of some recurring conformations from free protein ensemble (Heller et al., 2015; Ban et al., 2017). In latter case, the drug preferentially binds to some suitable conformations of free protein. Searching for such recurring conformations can provide transient binding pockets to design drugs that fit into them and bind favourably. For example, computational analysis of NMR and Small Angle X-ray Scattering (SAXS) can generate conformational ensembles of IDPs, which would contribute to identifying pharmacological cavities and calculated properties related to their shape and size (Yuan et al., 2013). A small variance in the surface area and volume of the cavities within an ensemble could be detected, while the difference in the average values is considerable between different ensembles. The cavities in IDPs could have a larger surface area, volume, and cavity depth, as compared to those in folded proteins, suggesting the possibility of strong ligand binding. Cavities can be clustered based on their average position along the sequence (determined by taking an average of the positions of the atoms constituting the cavity along the sequence of the IDP), and parameters determining the degree of conservation can be calculated for each cluster. The shape conservation of the cavities in each cluster can be measured from the standard deviation of the cavity depth and figure factor. The compositional conservation could be determined by calculating the percentage of atoms common between the cavities in a cluster, which is found to be very high (>50% for many clusters), suggesting that the shift of potentially pharmacological cavities among conformations is small and that the druggable cavities are conserved among different conformations, thus presenting the possibility of a rational drug design approach. The cavities could be found to have a high binding affinity toward test ligands.

## Conclusion

The mechanisms involved in CRPC progression is multifactorial, and complex molecular events underlying resistance to current drugs is incompletely understood. At present, the full-length AR has been a validated drug target, and LBD is the direct target for currently FDA-approved drugs. The resistance to these drugs involves the expression of constitutively active AR-Vs that lack the folded LBD. AR is unique from other steroid hormone receptors in that no transcriptional activity is attributed to its LBD, but rather transcriptional activity largely resides in NTD. Therefore, the NTD provides an attractive target as a drug binding to specific regions in this domain should be efficacious to block the transcriptional activation of all AR species, including full-length AR, AR-Vs and ARs with gain-of-function mutations in the LBD. However, NTD is intrinsically disordered protein that are currently inaccessible to conventional structure-based drug-design methods utilized for folded proteins. This disordered structures can have multiple and changing conformations thereby creating a challenge to discover drugs. Fortunately, the clinical translations of drugs can also depend on coherent pharmaceutical research based on biologically accurate screening approaches. Since establishing the cell culture method *in vitro*, our scientific community has improved cell-based drug screening assays and models. Moreover, the recent advances result in more informative biochemical assays and the development of highly accurate protein structure prediction, which potentially contributes the development of NTD-inhibitors screening. To date, there has been success with some small molecules directly binding to NTD, of which some have reached clinical trials. Overall, these novel emerging inhibitors that target alternative modular domains of the NTD have a promising prospective for producing even more successful outcomes for patients either as monotherapy or in combination with other therapies, with the ultimate aim of reducing AR signalling unachievable by current therapies.

## Figures and Tables

**Table:** 

**(N/C interaction)**	amino terminal/carboxyl terminal interaction
**(AF-1)**	activation function-1
**(AF-2)**	activation function-2
**(ALT)**	alanine transaminase
**(ADT)**	androgen deprivation therapy
**(AR)**	androgen receptor
**(ARE)**	androgen response element
**(ARI)**	androgen receptor inhibitor
**(AST)**	aspartate transaminase
**(BADGE)**	Bisphenol A Diglycidic Ether
**(CRPC)**	castration resistant prostate cancer
**(choline kinase alpha)**	CHKA
**(CT)**	circuit topology
**(CARM1)**	co-activator-associated arginine methyltransferase 1
**(CBP)**	CREB-binding protein
**(cryo-EM)**	cryo-electron microscopy
**(DHT)**	dihydrotestosterone
**(DBD)**	DNA binding domain
**(DLTs)**	dose limited toxicities
**(ENT2)**	equilibrative nucleoside transporters 2
**(GR)**	glucocorticoid receptor
**(Hsp)**	heat shock protein
**(IDP)**	intrinsically disordered protein
**(IDR)**	Intrinsically disordered region
**(Kallikrein Related Peptidase 3)**	KLK3
**(LBD)**	ligand binding domain
**(LLPS)**	liquid–liquid phase separation
**(MTD)**	maximal tolerated dose
**(MD)**	molecular dynamics
**(MoRF)**	molecular recognition region
**(NTD)**	N-terminal domain
**(NMR)**	nuclear magnetic resonance
**(PSA)**	prostate specific antigen
**(PCa)**	prostate cancer
**(PROTAC)**	proteolysis-targeting chimera
**(SARDs)**	selective AR degraders
**(SLiMs)**	short linear motifs
**(scFv)**	single-chain variable fragments
**(SINT1)**	Sintokamide A
**(SAXS)**	Small Angle X-ray Scattering
**(SBMA)**	spinal and bulbar muscular atrophy
**(AR-Vs)**	splice variants of AR
**(SRCs)**	steroid receptor co-activator proteins
**(SPR)**	surface plasmon resonance
**(TFIIF)**	transcription factor IIF
**(Tau-1)**	transcriptional activation unit-1
**(Tau-5)**	transcriptional activation unit-5
**(UGT2B)**	UDP-glucuronosyltransferase
**(USAN)**	United States Adopted Names
**(WT)**	Urolithin (UroA), wild type
